# Alcohol*Read at Palestine meeting of the East Texas Medico-Chirurgical Society, November, 1902.

**Published:** 1903-06

**Authors:** E. E. Guinn

**Affiliations:** Rusk, Texas


For Texas Medical Journal.

### Alcohol.*

BY E. E. GUINN, M. D., RUSK, TEXAS.

*Mr. President and Gentlemen:*

Your chairman has assigned me a subject which is Alcohol, and as this paper is listed on section of *General Medicine,* I presume I am to give it a general application.

Alcohol is composed of carbon, hydrogen and oxygen, and is represented by the chemical formula C4 H6 O2. In speaking of alcohol in this paper, it is in a general way, meaning all alcoholic

*Read at Palestine meeting of the East Texas Medico-Chirurgical Society, November, 1902.

spirits, which differ extremely as to the per cent of alcohol contained. Alcohol in its strongest distilled form contains 10 per cent of water, but there is a legal standard of strength of spirits which is called "proof," and which consists of more water than alcohol, viz.: About 5721/100 volumes of water to 495/10 alcohol in 100 parts. The specific gravity of spirits of "proof" strength is about 12/13 of an equal volume of distilled water at 62° F.

Alcohol does not exist ready-found in nature, but is always the product of art. In the production of alcohol there are two processes required, by the one of which the component parts of vegetables are converted into alcohol, and by the other, the alcohol is separated from other compounds. The former is known as "fermentation" and the latter as "distillation." The essential compound out of which alcohol is made is sugar, and all saccharine substances may yield it. Sugar is a natural product and is found in nearly all vegetables, but it is also produced by chemical action from starch (which is much more abundant in nature than sugar). This chemical process is known as "fermentation." One pound of sugar fermented yields ½ pound of "proof" spirits, or about ¼ pound of absolute alcohol. The carbonic acid escapes as gas, but the alcohol remains in the water. This chemical process is set up by the addition of a fermenting yeast ("tornula cerevisiæ") to a solution of sugar in water, and maintained at a temperature of about 80° F. Those substances which contain sugar, in the least admixture of starch, are—besides sugar and treacle—the juices of plants, such as sugar-cane, sugar-grass, beet-root and parsnip; the sugar maple, the palm and fruits of all kinds, including the grape, from which alcohol was very early obtained. But as great as these sources are, they are quite insufficient to meet the wants of the market, and we are, therefore, thrown a step backwards and must select those substances which contain starch largely, and afford it cheaply; such as grain of all kinds: corn, barley, oats, wheat, rye, rice, millet, etc., etc. The starch in the grain is first converted into sugar, ferments are added, such as diastase or glycogen, which is itself a compound between starch and sugar. It is found in malt, and is supplied principally from barley. One hundred pounds of corn will produce 3½ gallons of proof spirits. One bushel of malt will produce 2 to 2½ gallons of spirits. One hundred pounds of starch is equal to 70 pounds of sugar, and will produce about 8 gallons of proof spirits. However, this spirit is not all pure; the last of the distillation contains various essential oils: fusel oil largely predominates in the last of the distillation or the last part of the spirits that passes over. The best and purest spirits are obtained by a second distillation.

Spirits containing fusel oil should not be drank, but should only be used in the preparation of varnishes, etc., but it is a fact that it is used in adulteration of spirits, and is deleterious to health. Spirits of wine has a natural taste, and is not used as ardent spirits; however, it forms a basis of all ardent spirits.

America produces the greatest quantity of whiskey of any country in the world; most all (if not every one) of the States and territories in the Union are said to be producers, and especially Pennsylvania, Kentucky, Ohio, Illinois, Indiana and New York. The States of Pennsylvania and Kentucky are said to make the best whiskeys.

Cheap whiskey is rough and fiery, quickly intoxicates, and readily produces diseases of the mucuous membranes of the stomach, as well as the liver, spleen and kidneys. (The degradation and extermination of the Red Indians are attributed to mean whiskey.)

It is difficult to fix upon a standard of whiskey for general use, for the varieties are many, and hence uniformity of action from different specimens of the fluid can not be expected. Whiskey, in medical doses, generally decreases the amount of carbonic acid given off by the lungs, lowers respiration and accelerates the pulse. Brandy is probably preferable to any other spirits. It should be prepared by distillation from wine. One thousand gallons of wine distilled should yield from 100 to 150 gallons of brandy, consisting of about 50 to 54 per cent of absolute alcohol. The most of brandy, however, is made from alcohol and contains no wine.

### FOOD.

Various kinds of alcoholic liquors are used and considered, by many, as food, and I am sorry to say many physicians recommend them as food, or digestants. Perhaps beer or malt is the most used for this purpose. Of ardent spirits, rum alone exhibits the action of food, while gin, brandy and whiskey only lessen vital action; and the whole class disturbs the vital action, prevent a uniform course of change, and have much more the character of a medicine than a food and thus the tendency to lessen the action of all classes of those members which abound in alcohol; or in other words, to dry the skin—a tendency which is as marked as the effect of the spirit upon the sensorium and vital actions. Thus the hands and feet, and skin generally, become hot and dry. With such an action (which, however, is universal in reference to the elimination of water from the body) other results follow. The cooling of the body is lessened by the diminution in the quantity of fluid emitted by the skin, which is converted into vapor, with an enormous absorption of latent heat. The blood is diverted from the circumference towards the center so that the pulse becomes fuller and harder, and the liver and other large circulation centers receive more blood. This tendency is to increase vital processes and central nutrition, and in the constant user, or food advocate, from this extra nutritive, or blood supply, to these central organs—such as the stomach, liver and kidneys—we can readily see the ultimate result of this so-called food. A food should *nourish* and *preserve* the *body;* give it *endurance* and *strength.*

This is not the case with the alcohol user. The habitual user can not cope with the ravages of typhoid or pneumonia—a fact very evident to every physician in this society—therefore, can we assume that alcohol is a food in any form? The action upon the sensorium and nervous centers clearly depends upon the quantity of alcohol which is taken in a given time, other things being equal. When there is a perceptible effect, or an approach to it, there is relaxation of the animal-tissues, and particularly the muscles, so that contraction is less easily and fully effected, and the capacity to continue exertion is lessened. In a state of health, the tendency of alcohol is to diminish muscular power. The action of alcohol is opposed to that of food (which increases muscular power) and is, therefore, not a food.

The *Literary Digest* says "those who do not like to give up their alcoholic beverages are fond of reminding us that there may be intemperance in eating as well as drinking." Candy comes in for special condemnation. To decide this issue, it says, "a certain Frenchman made the following experiment: He fed a dog for fifty-four days on meat and sugar, the dog running thirty-four miles daily while undergoing the experiment, and gained during this time 1/20 of his weight; when $\frac{1}{3}$ of the sugar ration was replaced with alcohol, the dog's condition began to grow poor; he had to be urged to run by his trainer, although he had lost only an ounce or two of weight. Two rations (sugar and alcohol) were then alternated, each being used for a week, and the corresponding gain and loss of energy were evident." The investigator concludes that "alcohol is not a strength-giving ration."

In the Boer army alcoholic drinks are prohibited. The *Literary Digest,* quoting from an African paper, says "liquor drinking has been prohibited in our army. Although our men have been in the saddle hundreds of miles at a stretch, and in all sorts of weather, without warm overcoats, etc., but none of them have caved in." The heat nor cold did not hurt the men. It further states that "their medical men attribute this remarkable endurance to the non-use of alcohol." And further states that a "company of a few comrades of Boers were without food or drink all day; at dark one of the men found, in an abandoned farm-house, a bottle of brandy, of which they all drank except one, and all who drank were in a raging fever within half an hour. All the hunger and thirst that this writer had experienced during the campaign did not equal the ill effects of this one drink of brandy!" And he further states, "had we met the British, there was only one of us able to fight, the one who refused to drink." He further says: "It is absolutely false that alcohol raises the courage; the only result it has is to make the men more careless. It may have been of some value in earlier days, in hand-to-hand fighting, but what is wanted today is iron nerve, a clear eye and quick decision. Alcohol gives the reverse."

The correspondent of *Modern Medicine* says: "Alcoholic drink makes the drinker feel warm while really it lowers his temperature."

Dr. Victor Horsley, in *Literary Digest* says: "By signal test it was shown that alcohol blunted ideation—requiring longer time for forming same, though the subject thought he was catching the signal faster. It is a bed of deception." He further found that it was impossible to lift a given weight, while using alcohol, without tremor, showing its weakening effects upon the muscles. Even moderate doses of alcohol produced this condition, which was not present when alcohol was not used."

A food should promote both mental and physical power; therefore—and from other reasons too numerous to mention—I say that alcoholic beverages are not foods, and should never be recommended as such by physicians. We all know what the family physician says is generally accepted as true; then, Doctors, if you prescribe the use of alcoholic drinks as food or medicine, and your patient fills a drunkard's grave, who is responsible for it?

Now we will look at alcohol as a therapeutic agent:

First, Bartholow groups alcohol with the narcotic list. He recommends it in small doses as a stomach tonic. Certainly a very dangerous recipe! Let's use in its stead Tr. Nux Vomica, Gentian or Columbo; these agents will readily fill this place. He also recommends it in summer diarrhea, both in infants and adults. I have never seen any good results from its use in these conditions. Brandy is used with ice in vomiting, especially in pregnancy; here it has always failed with me. I had rather rely upon the ice alone. Its use is also recommended before an anesthetic. It is also used to counteract the depressing influence of aconite, veratrum, digitalis and snake bites. In the former case do not use it except perhaps in shocks, and then I would prefer morphine and atropine. In the latter I also think we could do better; ammonia would act as quickly, followed up by strychnine, etc., and these do not depress the general vitality as does alcohol. "In depressing maladies, such as acute fevers and inflammations, it should be used to give power to the heart muscles," says Bartholow. I consider that its use is positively contradicted in disease of this nature, and to convince any one of the fallacy, take one or two ounces (the usual medical dose), and while I admit for the 20 or 30 minutes after taking it you will feel bouyant (which is due to its action upon the sympathetic nervous system, and the superficial capillaries are engorged as well as those of the brain), but this soon passes off, and the depression sets in and you feel weakened all over, especially the muscular system; therefore it does not contract the heart muscle, but weakens it, and a static or engorged condition of the capillary system is the effect. In a weak heart, there are only two ways to stimulate it, one through the nervous system, and the other by increasing the blood volume; therefore, to stimulate the muscles of the heart, it is more lasting to do so through the nervous system, in acute and depressing diseases, and for this purpose strychnia, digitalin and atropia are preferred by me.

If, after some depleting disease, or injury, where the blood volume has been reduced, theoretically, intravenous or subcutaneous transfusion is indicated, and remedies calculated to throw the blood to the central organs. But permit me to digress and I will give you my experience with intravenous transfusion.

On the 14th day of July, I had a patient who presented himself with a severe lacerated wound in the region of Scarpa's triangle, produced by considerable force with the sharp end of a piece of timber. It left the common femoral artery and vein exposed, having deunded them of their covering, and the external coat of the femoral artery was injured. After thoroughly cleansing the wound, I closed it, leaving drainage. The man's temperature was

102° at the time and ran to 104° each afternoon. Eight days later, tissues over the surface of the spinous process of the illium became œdematous and red; the stitches were removed and the wound thoroughly washed out. At this juncture I was called away, and was absent four or five days, and the patient was left in charge of Dr. Wiggins, who, two days after I left, extended the incision from wound to the œdematous surface and evacuated considerable pus, and the pocket was packed. Next morning the deep external pudic ruptured and patient lost a great deal of blood. The doctor used subcutaneous transfusion of saline solution. Everything went well until the second day after I returned, when the patient had another very severe hemorrhage (I had dressed the wound the day before; it was clean and granulating and not a drop of pus), I threw a rubber bandage around the wound and leg, and this controlled the hemorrhage. With the assistance of Dr. R. Y. Lacy, we opened up the basilic vein in the left arm and gave him 24 ounces of saline solution without any perceptible change in either his pulse rate or volume. Next day I dressed the wound and discovered that the femoral vein had ruptured and caused the hemorrhage. The artery was granulating. I had no hemorrhage while dressing the wound, which was clean and nice; I re-packed very closely with gauze and put on a snug rubber bandage. All went well till the socond day following, when I was present, talking to the patient; he was perfectly quiet, and the hemorrhage began running from under the dressing in great quantities and was arterial blood. I immediately threw a rubber bandage around the leg, but this failed to control the hemorrhage; I took the scissors and rapidly cut off all dressing, while an assistant made pressure over the femoral artery above, which, however, did little good. As soon as the dressing was off, caught the artery with my fingers and held same while Dr. Lacy ligated it with silk.

We again used intravenous transfusion, this time in vein of right arm, using 40 ounces, as he had apparently lost this amount of blood, but there was absolutely no effect upon the pulse, and he had never regained the color in his lips from the first hemorrhage. His fever had never abated; I had kept him nourished on concentrated foods from the first hemorrhage, and his appetite was good. Two days later he died. Autopsy showed the blood vessels to be in the condition described.

The point is, did the intravenous transfusion really do any good? Perhaps it prolonged his life a few days, but I saw no immediate effects from its use. The patient of course was not anesthetized, and described his feeling as if the saline solution was running freely into his brain and general system; said it was filling him up, and made him feel better and stronger.

Now again to alcohol: I could define its use as a medicine from various authors, but this you can all read from your text-books and magazines. I believe we could discard its use altogether as a therapeutic agent, but at the present time we must admit its usefulness in the preparation of various chemical compounds. It is used in the process of various laboratory and microscopical procedures, which, no doubt, could be replaced by some other agent. I

believe it would be a positive chemical antidote to carbolic acid poisoning, if administered at once. It absolutely antagonizes the effects of carbolic acid locally where it is at once applied.

My intentions were to discuss its effect from a moral standpoint, but I will not attempt this time to enter upon this, the most vital point of this subject. In conclusion, I say that alcohol is not a food, and as a medicine its evil effects upon the system and the danger of its immoral effects upon the patient, and the world at large, should never be forgotten, and its use, therefore, resorted to only when absolutely necessary, and when no substitute will answer.

